# Solution-Processable Cu_3_BiS_3_ Thin Films: Growth Process Insights and Increased Charge Generation Properties by Interface Modification

**DOI:** 10.1021/acsami.3c10297

**Published:** 2023-08-25

**Authors:** Thomas Rath, Jose M. Marin-Beloqui, Xinyu Bai, Astrid-Caroline Knall, Marco Sigl, Fernando G. Warchomicka, Thomas Griesser, Heinz Amenitsch, Saif A. Haque

**Affiliations:** † Department of Chemistry, Imperial College London, Molecular Sciences Research Hub White City Campus, Wood Lane, London W12 0BZ, U.K.; ‡ Institute for Chemistry and Technology of Materials, NAWI Graz, Graz University of Technology, Stremayrgasse 9, 8010 Graz, Austria; § Institute of Materials Science, Joining and Forming, Graz University of Technology, Kopernikusgasse 24, 8010 Graz, Austria; ∥ Institute of Chemistry of Polymeric Materials, Montanuniveristät Leoben, Otto Glöckelstrasse 2, 8700 Leoben, Austria; ⊥ Institute of Inorganic Chemistry, NAWI Graz, Graz University of Technology, Stremayrgasse 9, 8010 Graz, Austria

**Keywords:** metal sulfides, precursor chemistry, X-ray scattering, interface, transient absorption spectroscopy

## Abstract

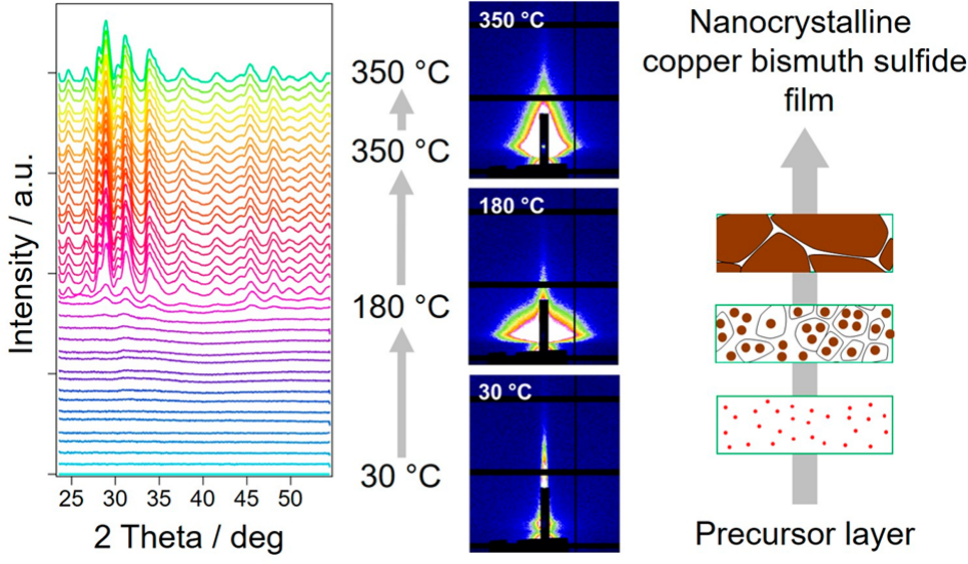

Cu_3_BiS_3_ thin films are fabricated via spin coating of precursor solutions containing copper and bismuth xanthates onto planar glass substrates or mesoporous metal oxide scaffolds followed by annealing at 300 °C to convert the metal xanthates into copper bismuth sulfide. Detailed insights into the film formation are gained from time-resolved simultaneous small and wide angle X-ray scattering measurements. The Cu_3_BiS_3_ films show a high absorption coefficient and a band gap of 1.55 eV, which makes them attractive for application in photovoltaic devices. Transient absorption spectroscopic measurements reveal that charge generation yields in mesoporous TiO_2_/Cu_3_BiS_3_ heterojunctions can be significantly improved by the introduction of an In_2_S_3_ interlayer, and long-lived charge carriers (*t*_50%_ of 10 μs) are found.

## 1 Introduction

The copper bismuth sulfide Cu_3_BiS_3_ (wittichenite) is an attractive material for photovoltaics but has scarcely been explored regarding solar cell applications up to now.^1−6^ Cu_3_BiS_3_ has an orthorhombic crystal structure^7^ and is one of the 13 copper bismuth sulfide phases which are stable at room temperature.^8^ It is a p-type semiconductor and has a band gap of 1.5–1.6 eV ^9^ and a high absorption coefficient of more than 10^5^ cm^–1^, and the charge carrier density in Cu_3_BiS_3_ can reach 2 × 10^16^ cm^–3^.^8^ Moreover, bismuth is significantly less toxic than the elements used for photovoltaics which are situated around bismuth in the periodic table. In addition, bismuth and also copper have a high abundance in the earth crust, which makes copper bismuth sulfide a relatively inexpensive material.^10^

First Cu_3_BiS_3_ based thin film solar cells with power conversion efficiencies up to 0.29% have been reported recently^3−6^ and by Ag doping the efficiencies could be increased to 0.48%.^5^ Dhanak et al. thoroughly studied the Cu_3_BiS_3_ electronic structure and band alignment regarding photovoltaic applications;^11^ however, investigations on charge generation yields and charge carrier lifetimes have not been in the focus so far. Yin et al. investigated Cu_3_BiS_3_ in a photoelectrochemical cell and obtained a power conversion efficiency of 1.28%.^12^ Furthermore, applications of Cu_3_BiS_3_ in photoelectrochemical hydrogen production,^13−15^ lithium-ion batteries,^8^ photothermal therapy,^16,17^ photothermal conversion,^18^ and thermoelectrics^19^ have been explored.

There are several reports on the synthesis of Cu_3_BiS_3_ nanoparticles via colloidal^9,17,20^ or solvothermal approaches,^21,22^ and preparation routes for Cu_3_BiS_3_ thin films have been reviewed by Deshmukh and Kheraj.^23^ Cu_3_BiS_3_ films have been prepared via (reactive) sputter deposition,^24,25^ chemical bath deposition,^8,26−28^ electrodeposition followed by sulfurization,^29,30^ or coevaporation.^31,32^ Reports on solution-based thin film deposition approaches beyond chemical bath deposition are quite limited. So far, spray pyrolysis^33^ and coating of nanoparticle inks^9^ have been used for thin film preparation, and precursor routes based on copper and bismuth salts and sulfur sources such as thiourea^3,6^ or based on CuO and Bi_2_S_3_ in thiol–amine solvents mixtures have been investigated.^34^

In this paper, we introduce an alternative solution-based synthesis method for nanocrystalline Cu_3_BiS_3_ thin films. In this approach, metal xanthates are used as precursors, which are known to be versatile single-source metal–organic precursors for the formation of various metal sulfides. They are soluble in many solvents^35^ and are converted to metal sulfides at comparably low temperatures (140–210 °C)^36^ and even at room temperature using UV-light illumination,^37^ while they have a shelf life of several years when stored in ambient conditions. The low conversion temperature, simple synthesis of the metal xanthates, and convenient processing make this approach highly desirable for the preparation of solar cells.

For solar cell applications, metal xanthates have been applied for the fabrication of polymer/nanocrystal bulk heterojunction absorber layers in which the metal sulfide nanocrystals were directly synthesized within the polymeric matrices.^38−42^ Furthermore, they have been used for the fabrication of semiconductor sensitized solar cells as metal xanthate precursor solutions can well infiltrate mesoporous metal oxide scaffolds and cover them with thin metal sulfide films after thermal annealing.^43,44^ Moreover, the application of copper zinc tin sulfide (CZTS) films prepared from metal xanthates has been already shown to be suitable for the application in thin films solar cells.^45^

In this study, we extend this approach to the design, synthesis, and characterization of copper bismuth sulfide Cu_3_BiS_3_ (CBS) films for the application in semiconductor sensitized solar cells. More specifically, we present a detailed structural and optical characterization of the Cu_3_BiS_3_ thin films by X-ray diffraction, Raman spectroscopy, SEM-EDX measurements, and UV–vis spectroscopy. In addition, we report on the investigation of the formation of the Cu_3_BiS_3_ nanocrystalline films by a combined time-resolved grazing incidence small and wide angle X-ray scattering (GISAXS, GIWAXS) study using synchrotron radiation. Furthermore, we studied the photoinduced charge transfer kinetics in mesoporous TiO_2_/Cu_3_BiS_3_ films by transient absorption spectroscopy and investigated the effect of an In_2_S_3_ interlayer on these kinetic processes. These findings indicate efficient charge transfer in mesoporous TiO_2_/Cu_3_BiS_3_ heterojunctions with an In_2_S_3_ interlayer, which makes such heterojunctions attractive for solar energy conversion.

## 2 Results and Discussion

**Figure 1:**
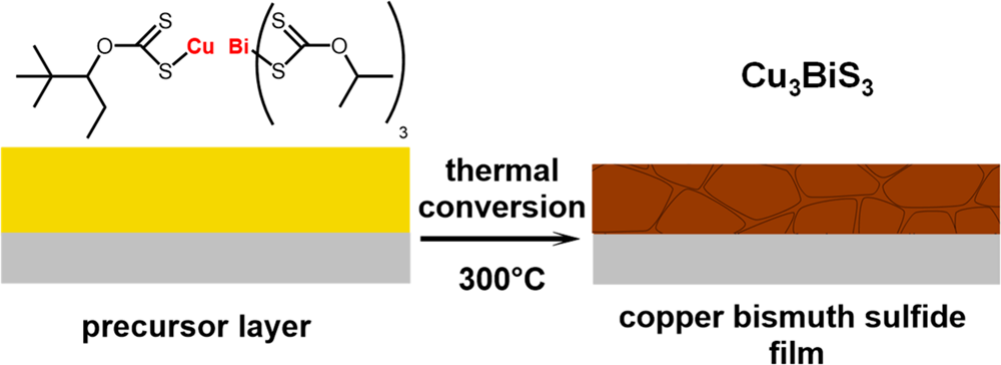
Schematic illustration of the formation of Cu_3_BiS_3_ thin films from copper and bismuth xanthates (the gray layer represents a substrate).

Figure 1 illustrates the solution-based method for the preparation of Cu_3_BiS_3_ films reported herein. In the first step, copper xanthate and bismuth xanthate are dissolved in a molar ratio of 3:1 in chlorobenzene. Details of the synthesis of the copper and bismuth xanthates and the preparation of the precursor solution are given in the Experimental Section of this paper. The combination of copper 2,2-dimethylpentyl xanthate and bismuth isopropyl xanthate was selected, as this combination gives stable precursor solutions in chlorobenzene/pyridine (93/7 vol/vol) as solvent. Pyridine acts as an additional ligand for the metal ions in the solution. The use of copper xanthates with shorter, nonbranched side chains would not be possible, as they are not soluble in chlorobenzene.^38^ Using bismuth xanthates with shorter nonbranched alkyl moieties, e.g., bismuth ethyl xanthate, would lead to precipitation of the copper xanthate in the chlorobenzene solution.

**Figure 2:**
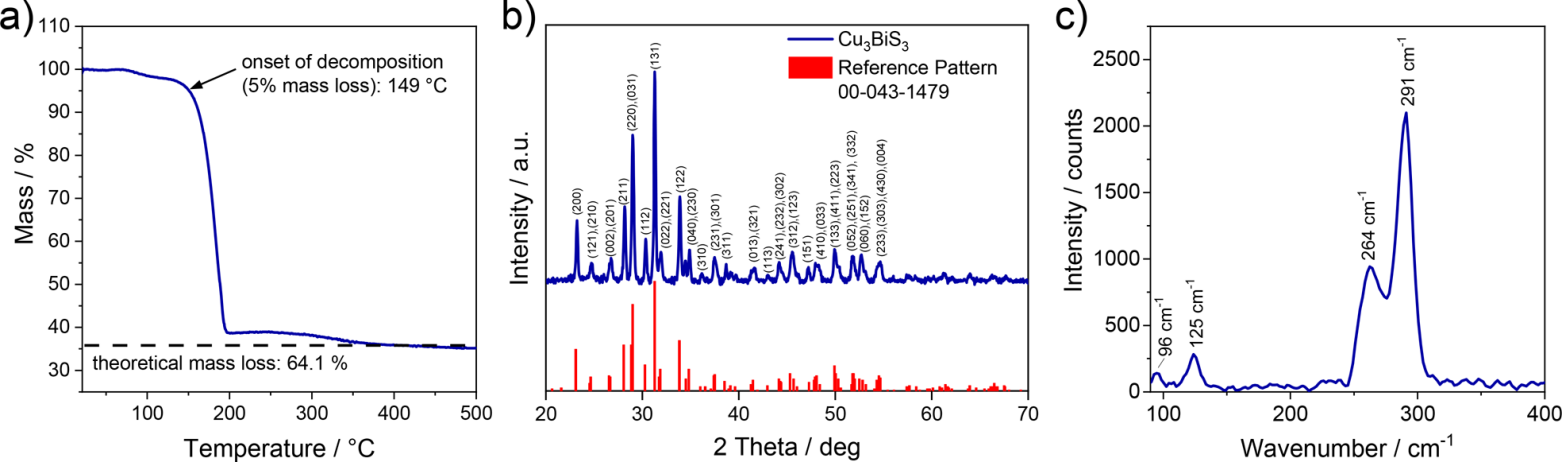
(a) Thermogravimetric analysis of the conversion of copper and bismuth xanthates to Cu_3_BiS_3_ and an X-ray diffraction pattern (b) as well as a Raman spectrum (c) of Cu_3_BiS_3_ prepared at 300 °C.

Next, the precursor solution is spin coated on the substrate followed by a heating step at 300 °C. The absorption spectrum of the pale yellow precursor film is shown in Figure S1, and Videos S1 and S2 of the thermal conversion of precursor films (spin coated and drop coated) to Cu_3_BiS_3_ films are included as supporting material. Thermogravimetric analysis (see Figure 2a) shows that the metal xanthates are stable up to a temperature of 149 °C. At this temperature, they start to decompose following a Chugaev elimination reaction, whereby the Cu_3_BiS_3_ phase is formed and volatile decomposition products (COS, CS_2_, and corresponding alkenes)^38^ evaporate from the film. The measured weight loss is identical with the theoretically expected value of 64.1%, indicating complete conversion of the metal xanthates to Cu_3_BiS_3_. The X-ray diffraction pattern in Figure 2b confirms the formation of an orthorhombic wittichenite copper bismuth sulfide, and the measured pattern agrees with the reference pattern. We note that no secondary phases are observed. The formation of Cu_3_BiS_3_ is further confirmed by Raman spectroscopy. The Raman spectrum shows two main peaks at 264 and 291 cm^–1^ as well as two minor ones at 96 and 125 cm^–1^, which are characteristic for wittichenite Cu_3_BiS_3_.^11,25^ The chemical composition analysis of the synthesized Cu_3_BiS_3_ thin films with scanning electron microscopy energy dispersive X-ray spectroscopy (SEM-EDX) reveals that the composition of the ternary metal sulfide is close to the theoretically expected one (see Table 1 and Figure S2 for the corresponding EDX-spectrum). Additional XPS measurements (Table 1, Figure S3) support the findings from the SEM-EDX measurements.

**Table 1:** Chemical Composition of Cu_3_BiS_3_ Obtained from SEM-EDX and XPS Measurements

material		Cu, atom %	Bi, atom %	S, atom %
Cu_3_BiS_3_	SEM-EDX	43.5 ± 0.2	12.3 ± 0.1	44.2 ± 0.2
	XPS	41.8	14.4	43.8
	theoretical	42.86	14.28	42.86

The formation of the Cu_3_BiS_3_ films from the copper and bismuth xanthate precursor films was thoroughly investigated by time-resolved simultaneous grazing incidence wide and small-angle X-ray scattering (GIWAXS and GISAXS) measurements using synchrotron radiation. For this, the substrates coated with the precursor film were heated from room temperature to 350 °C with a heating rate of 10 °C/min in a nitrogen-flushed measuring cell. One measurement was acquired every 6 s, which equals a temperature resolution of 1 °C. Once the target temperature was reached, the sample was held for seven more minutes at this temperature.

**Figure 3:**
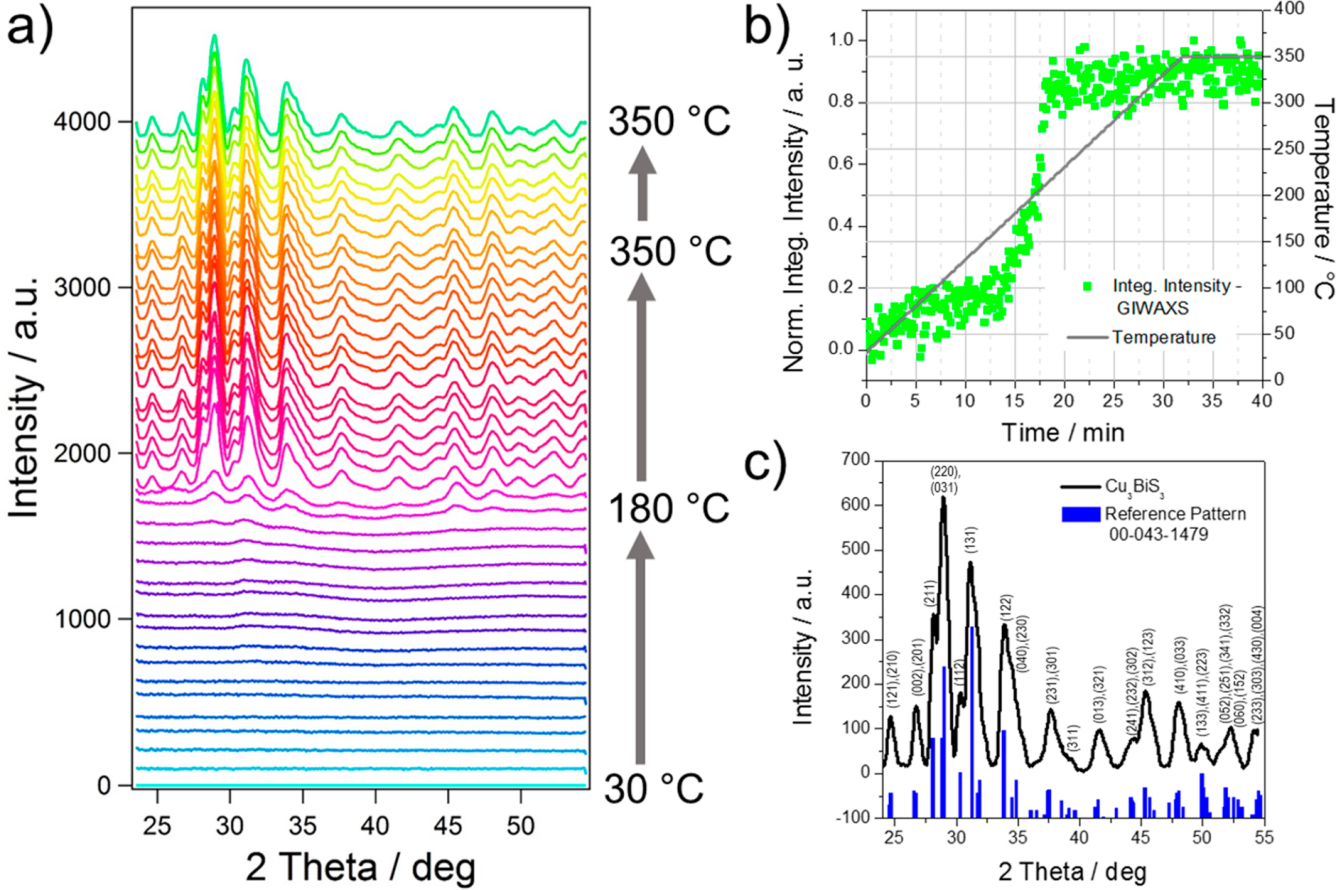
(a) Evolution of the GIWAXS pattern during the heating run from 30 to 350 °C (every 10th frame measured is shown), where the patterns were obtained by azimuthal integration in out-of-plane direction, (b) normalized integrated intensity of the GIWAXS patterns (integrated from 27.5 to 29.8° 2θ), and (c) the GIWAXS pattern measured at 350 °C with the corresponding reference pattern.

Figure 3a shows the evolution of the GIWAXS pattern with increasing temperature. Up to a temperature of 170 °C, no peaks are observed. At 180 °C, first indications for diffraction peaks are visible, which become much more intense within the next frames. The evolution of the diffraction peaks can also be visualized by the integrated intensity, shown in Figure 3b. Here, it can clearly be seen that the diffraction peaks start to evolve at a temperature of 175 °C and, at around 210 °C, the growth of the peaks stagnates, indicating that the formation of the Cu_3_BiS_3_ phase is completed at this temperature. In Figure 3c, the pattern measured at 350 °C is depicted together with a reference pattern for Cu_3_BiS_3_. Compared to the X-ray diffraction pattern (Figure 2b), the peaks in the GIWAXS pattern are distinctively broader due to the scattering of the X-rays at different positions over a larger sample area in the grazing incidence setup; however, the GIWAXS pattern also coincides well with the reference pattern.

It should be also mentioned that based on the evolution of the GIWAXS patterns, neither crystalline copper sulfide intermediates, which are quite common in the synthesis of ternary copper chalcogenides,^9^ nor crystalline bismuth sulfide intermediates were found in this conversion process using metal xanthates as precursors.

**Figure 4:**
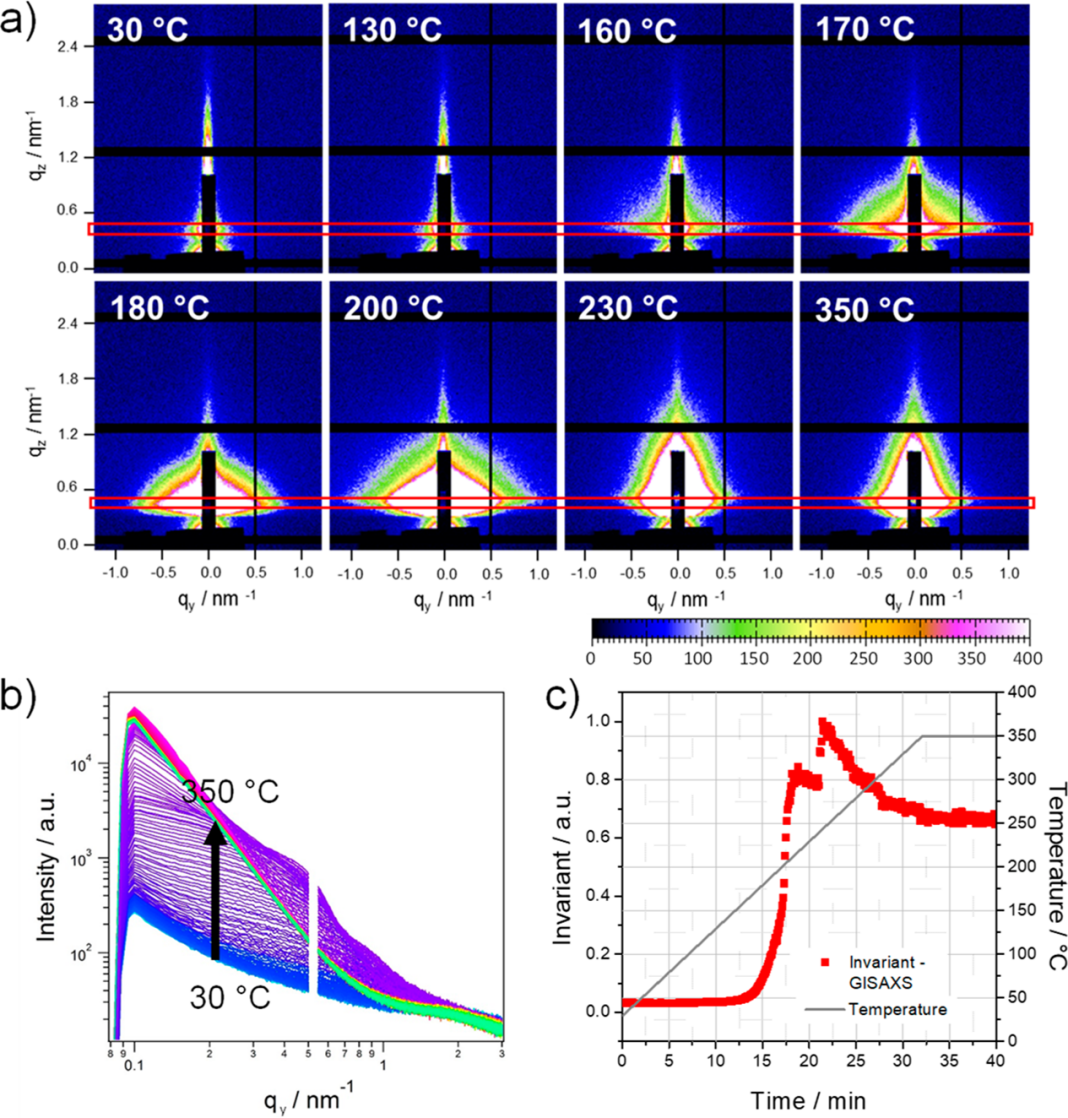
(a) GISAXS images of the sample at different temperatures during the formation process of Cu_3_BiS_3_ (the red box in the GISAXS images indicate the areas used for horizontal integration at *q*_*z*_ = 0.50 nm^–1^), (b) evolution of the in-plane scattering signal (the arrow indicates the temperature increase during the heating run from 30 to 350 °C indicated by the color change of the curves from blue to violet, pink, and green), and (c) the corresponding invariant data of the GISAXS curves integrated from 0.1 to 1.3 nm^–1^ in *q*_*y*_ direction.

GISAXS data, acquired simultaneously with the GIWAXS measurements, provide further insights into the Cu_3_BiS_3_ formation process. In Figure 4a, GISAXS patterns measured at selected temperatures during the heating run are depicted. They demonstrate a strong increase of scattering with increased temperature due to the conversion of the metal xanthates to Cu_3_BiS_3_. The formation of the metal sulfide leads to a significant increase in electron density in the film, as the organic moieties evaporate from the film during the conversion step. The areas used for horizontal integration are marked with a red box in the GISAXS images in Figure 4a, and the resulting cuts at *q*_*z*_ = 0.5 nm^–1^ during the heating run from 30 to 350 °C are presented in Figure 4b.

For a first analysis of the GISAXS data, we used the Porod invariant integrated over a limited *q*-range from the in-plane GISAXS data as a sensitive qualitative measure of the changes in the thin films during the heating run. Details regarding the calculation of the Porod invariant are given in the Supporting Information. The evolution of the Porod invariant of the GISAXS curves (calculated between *q*_*y*_ = 0.1 and 1.3 nm^–1^), which is shown in Figure 4c, reveals that minor structural changes are already occurring in the metal xanthate film, starting at a temperature of around 150 °C, the temperature at which the metal xanthates start to decompose. A significant increase in the invariant was observed between 175 and 210 °C. This originates from the formation of the Cu_3_BiS_3_ nanocrystals in this temperature range, as revealed by the GIWAXS investigations. The slight increase of the invariant at approximately 240 °C originates most likely from a compaction of the Cu_3_BiS_3_ layer. In the further course of the heating run, the value of the invariant decreases again as the scattering intensity in the detected *q*-range decreases due to the agglomeration of the nanocrystals and the formation of a continuous flat film. In addition, the GIWAXS images (Figure 4a) reveal no preferred orientation of the nanocrystals in the thin films with respect to the substrate as has been observed in the formation of nanocrystalline CuInS_2_ films from metal xanthates.^46^

**Figure 5:**
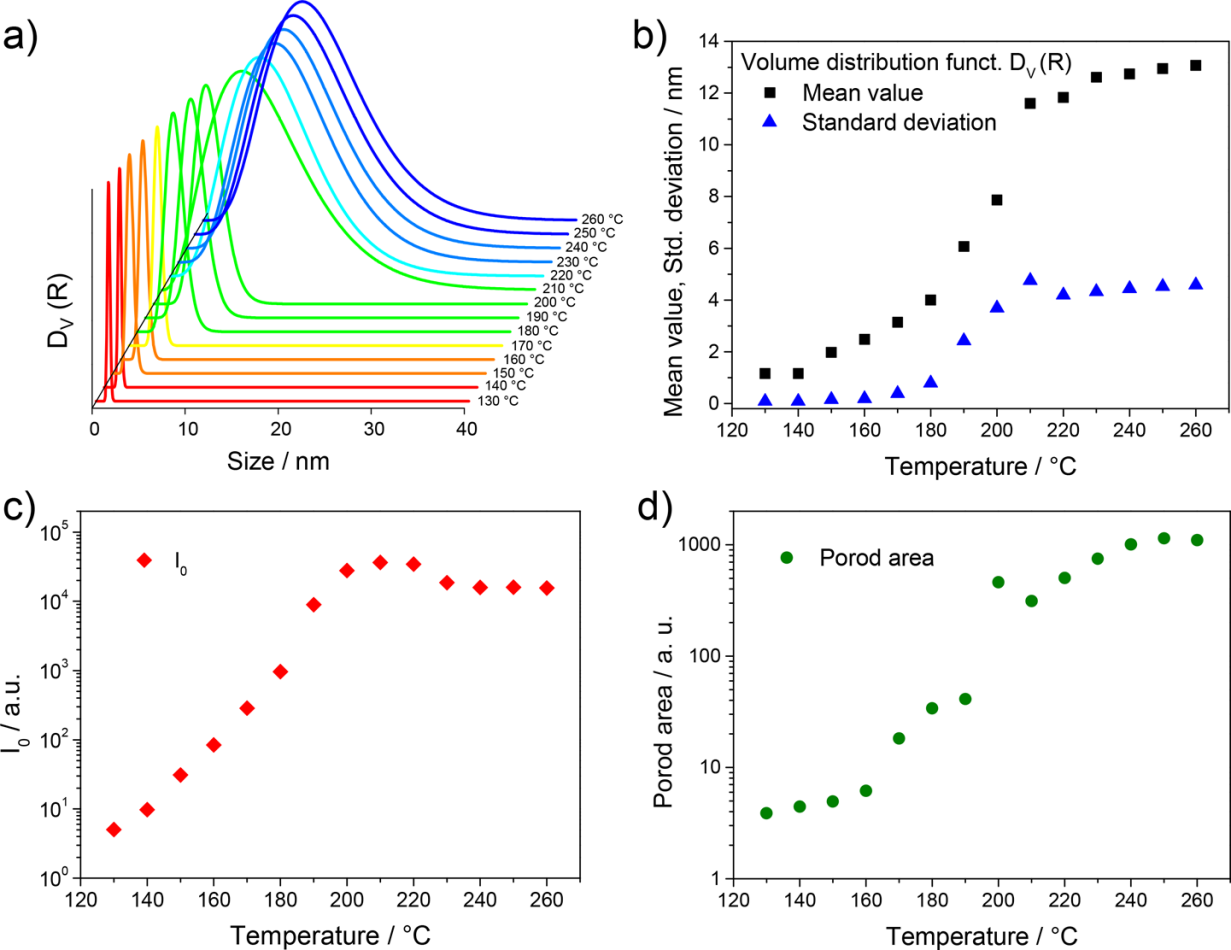
(a) Evolution of the volume weighted particle size distribution, (b) mean values and standard deviation of the particle size distribution, (c) scattering intensity *I*_o_, and (d) Porod area during the heating run.

Moreover, the GISAXS data were fitted by a simplified, analytical model rather than the full usage of the distorted wave Born approximation (DWBA) theory^47,48^ as we set the focus on relative changes in the samples during the heating run. Thus, the in-plane cuts of the GISAXS data were analyzed with a simplified structural model, including form factor scattering as well as a structure factor and a Porod contribution. More details about this model and the in-plane cuts together with the obtained fits are shown in the Supporting Information (Figure S4 and Figure S5). Based on the fit results, the particle sizes and particle size distributions in the film during the heating run could be determined. In Figure 5a,b, the development of the volume weighted particle size distribution function (*D*_V_(*R*)) and the corresponding mean values and standard deviations of the distribution functions are depicted. These results reveal that already at 130–140 °C, seeds with a diameter of approximately 1 nm are present in the film. As in this temperature range no diffraction peaks can be observed in the GIWAXS patterns, it can be furthermore concluded that they are amorphous. Between 140 and 180 °C, the mean value of the size distribution function (*D*_V_(*R*)) increases to 4 nm and the particle size distribution stays very narrow, indicating a monodisperse growth of the Cu_3_BiS_3_ nanocrystals up to a temperature of 180 °C. In addition, at this temperature the nanocrystals start to form clusters, and between 180 and 210 °C the mean value of the particle size distribution becomes significantly higher together with an increase of the standard deviation. In this phase of the conversion process, the organic matrix (xanthate moieties) has completely vanished from the layer, and the nanocrystals grow more randomly. Above 200 °C, the particle size distribution has a mean value of about 12 nm, with a standard deviation of 4 nm. The scattering intensity (depicted in Figure 5c) stays constant at temperatures higher than 200 °C (and even decreases slightly at temperatures higher than 220 °C), which indicates that the increasing size of the nanoparticles in this phase originates rather from agglomeration than further growth of the nanocrystals. This is also supported by the increasing Porod area (Figure 5d).

**Figure 6:**
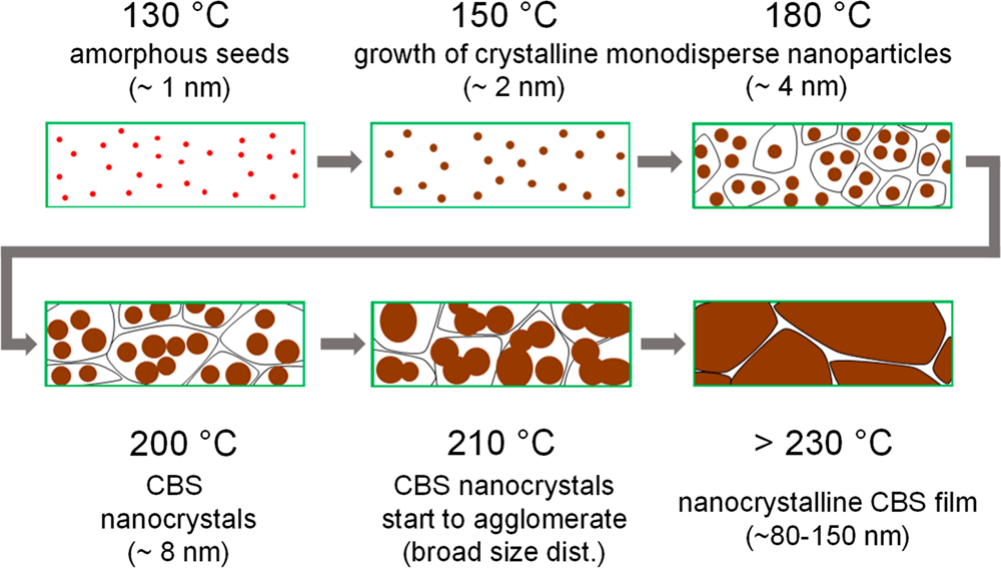
Schematic illustration of the Cu_3_BiS_3_ formation based on the time-resolved X-ray scattering data.

**Figure 7:**
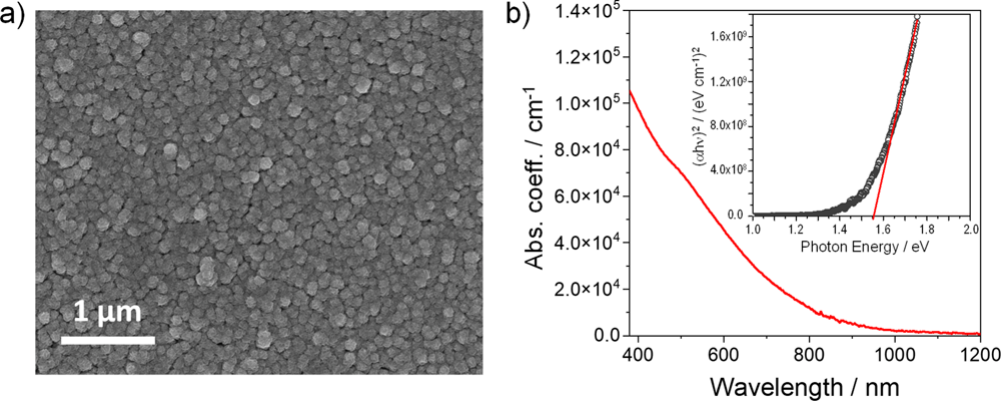
(a) SEM image of a Cu_3_BiS_3_ film prepared on glass and (b) UV–vis absorption spectrum of a Cu_3_BiS_3_ thin film annealed at 300 °C and the corresponding Tauc plot for band gap determination (shown in the inset).

Based on the GISAXS and GIWAXS data, a model for the formation of the Cu_3_BiS_3_ films can be proposed, which is illustrated in Figure 6. The process starts with small amorphous seeds (∼1 nm) at 130–140 °C, which become crystalline and grow continuously with increasing temperature up to a temperature of 180 °C, at which point highly monodisperse nanocrystals with a diameter of 4 nm are observed. Furthermore, in this temperature range, the nanocrystals already begin to cluster, as the organic matrix vanishes due to the decomposition of the metal xanthates and the evaporation of volatile decomposition products from the film. In the next phase of the growth process, the size distribution of the nanocrystals becomes broader and they start to agglomerate. At temperatures above 230 °C, the film formation is completed and grain sizes are too large to be detected with the GISAXS setup. To gather this information, we acquired a SEM image of a Cu_3_BiS_3_ film (Figure 7a) and grain sizes of about 80–150 nm were observed (Figure S6). Furthermore, to investigate the suitability of this precursor-based approach to prepare thicker films, we applied a simple drop coating process of the precursor solution followed by drying at room temperature and annealing at 350 °C, which resulted in a homogeneous nanocrystalline film with constant thickness of approximately 500 nm as revealed by cross sectional SEM images (Figure S7). To investigate the stability of the Cu_3_BiS_3_ films, we stored them in different conditions (room temperature and nitrogen atmosphere, room temperature and ambient conditions, 65 °C and nitrogen atmosphere) and analyzed the films via X-ray diffraction at certain times over a period of approximately two months. The corresponding diffraction patterns are depicted in Figure S8 and reveal no observable changes over time under all three storage conditions. In addition, pictures of the films after the stability tests are included in the Supporting Information (Figure S9).

Figure 7b shows the steady-state UV–vis absorption spectrum of a Cu_3_BiS_3_ thin film on a planar glass substrate. The film has an absorption coefficient up to 1 × 10^5^ cm^–1^ (at a wavelength of 400 nm), and the derived Tauc plot (inset) reveals a direct band gap of 1.55 eV, which is well suited for application in solar cells. The absorption coefficient and the optical band gap are well in line with the values obtained for Cu_3_BiS_3_ thin films prepared by other methods, as they are summarized in a review by Deshmukh et al.^23^ The review reports typical absorption coefficients up to approximately 1 × 10^5^ cm^–1^ and band gap values of 1.4–1.45 eV for Cu_3_BiS_3_ prepared via sputtering, electrodeposition, or coevaporation and slightly higher band gaps (1.55–1.72 eV) for films prepared via chemical bath deposition or spray pyrolysis.^23^

Next, we consider the optical and electronic properties of mp-TiO_2_/Cu_3_BiS_3_-based architectures typically used in semiconductor sensitized photovoltaic devices. To this end, nanocrystalline Cu_3_BiS_3_ thin films were prepared in one step directly on mesoporous (mp) metal oxide scaffolds without the need for capping ligands, which could impede or hinder charge separation or transport in photovoltaic devices. Moreover, this fabrication method allows the precursor solution to infiltrate into the mp-TiO_2_ film, thereby enabling a good coverage of the mp-TiO_2_ scaffold with the Cu_3_BiS_3_ absorber (see SEM image in Figure S10a). The absorption properties of the mp-TiO_2_/Cu_3_BiS_3_ films (Figure S10b) are very similar to Cu_3_BiS_3_ thin films prepared on planar glass substrates (Figure 7b).

**Figure 8:**
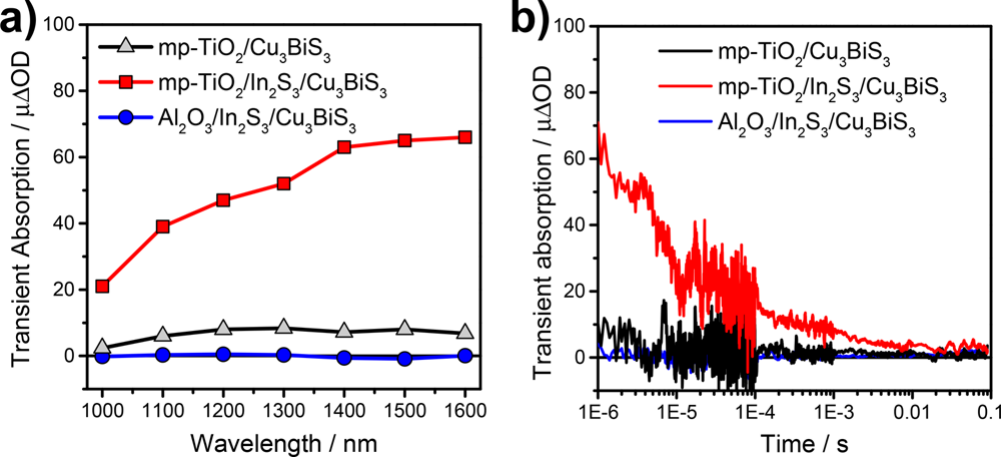
(a) Transient spectra recorded at 1 μs after exciting at 510 nm with a fluence of approximately 10 μJ/cm^2^ of mp-TiO_2_/Cu_3_BiS_3_ (black curve), mp-TiO_2_/In_2_S_3_/Cu_3_BiS_3_ (red curve), and Al_2_O_3_/ In_2_S_3_/Cu_3_BiS_3_ (blue curve); (b) TAS decay traces of mp-TiO_2_/Cu_3_BiS_3_, mp-TiO_2_/In_2_S_3_/Cu_3_BiS_3_, and Al_2_O_3_/In_2_S_3_/Cu_3_BiS_3_, where the samples were excited at 510 nm with a fluence of approximately 10 μJ/cm^2^ and probed at 1400 nm.

In order to study the charge carrier dynamics of the Cu_3_BiS_3_ films on an electron transport layer (mp-TiO_2_), microsecond to millisecond transient absorption spectroscopy (TAS) was used. This technique enables the identification of photogenerated charge carriers and monitoring of their decay dynamics. Briefly, the sample was excited by a nitrogen-pumped dye laser to obtain an excited state, which was probed by a second light source (a tungsten lamp). Transient spectra were measured at 1 μs following laser excitation at 510 nm with the same fluence for all of the samples (see Figure 8a). Charge recombination kinetics were traced by monitoring the fate of the transient absorption feature at 1400 nm. The steady-state absorption spectra of the investigated samples are depicted in Figure S11.

Figure 8a presents the transient absorption spectrum of the mp-TiO_2_/Cu_3_BiS_3_ (black curve), which exhibits a broad absorption feature between 1000 and 1600 nm characteristic of electrons in TiO_2_ or holes in metal sulfides.^43^^49^ This suggests that charge separation occurs at the mp-TiO_2_/Cu_3_BiS_3_ interface. This is most likely due to electron injection from the conduction band of photoexcited Cu_3_BiS_3_ to the conduction band of mp-TiO_2_. However, as can be seen in Figure 8a,b, the intensity of the signal is relatively low (around 10 μΔOD; ΔOD is the difference in optical density). Since the amplitude of ΔOD is closely related to the yield of charge generation, this indicates that despite the process of charge separation occurring, it most likely happens with a relatively low efficiency at the mp-TiO_2_/Cu_3_BiS_3_ interface.

The origins for the moderate charge separation yields at the mp-TiO_2_/Cu_3_BiS_3_ interface might be due to surface trap states at the grain boundaries, as has been shown previously.^31^ This hinders the development of efficient solar cells with a Cu_3_BiS_3_ absorber. A viable approach to diminish this effect would be the addition of a passivation layer to improve charge separation. In_2_S_3_ can passivate the surface trap states of Cu_3_BiS_3_ through a surface reaction of the two materials.^26^ As a consequence, the electronic structure of Cu_3_BiS_3_ and the distribution of the work function become much more uniform with this interlayer, as reported by Mesa et al.^50^

Therefore, to improve the efficiency of charge separation in the mp-TiO_2_/Cu_3_BiS_3_ layers, we explored the use of an In_2_S_3_ interlayer between the absorber (Cu_3_BiS_3_) and the electron acceptor (mp-TiO_2_). The In_2_S_3_ layer was prepared by spin coating of an indium xanthate precursor solution on mp-TiO_2_ followed by an annealing step at 350 °C. In a subsequent step, the Cu_3_BiS_3_ precursor solution was coated followed by a further annealing at 350 °C. A SEM cross section image of this mp-TiO_2_/In_2_S_3_/Cu_3_BiS_3_ sample is shown in Figure S12 and reveals a 1.5 μm thick mesoporous layer with no visible crystals or a compact film on top of the mesoporous film, indicating a good infiltration of the precursor solutions into the mesoporous TiO_2_ scaffold. An X-ray diffraction pattern of an In_2_S_3_ film (prepared via drop coating of the indium xanthate precursor solution and annealing at 350 °C) on glass is presented in Figure S13a, and the peaks match well with the reference pattern. Via SEM-EDX analysis (Figure S13b), an In:S atomic ratio of 42.5:57.5, and thereby a slightly sulfur poor composition, is found. Figure 8a,b shows that the presence of this interlayer significantly increases the transient absorption signal amplitude from approximately 10 to 60 μΔOD. Moreover, the similarity in the broad band signals of the transient absorption spectra of mp-TiO_2_/Cu_3_BiS_3_ (Figure 8a, black curve) and TiO_2_/In_2_S_3_/Cu_3_BiS_3_ (Figure 8a, red curve) samples is consistent with an enhancement in electron injection yield from Cu_3_BiS_3_ to the mp-TiO_2_. The higher charge generation observed in the samples employing an In_2_S_3_ buffer layer is in accordance with the expected role of In_2_S_3_. Passivation of Cu_3_BiS_3_ trap states enhances the charge transfer to TiO_2_ because charges are not trapped at the grain boundaries. Moreover, the presence of the buffer layer generates a band bending to the Cu_3_BiS_3_ conduction band, which makes the electron injection to the TiO_2_ more favorable.^26,31,50^ The recombination lifetime (*t*_50%_) was deduced by obtaining the time for 50% of the excited TiO_2_ to relax back to its ground state. In this way, we find that the charge carriers are relatively long-lived with a half-life (*t*_50%_) of around 10 μs.

Finally, to confirm that electrons are injected into the mp-TiO_2_ instead of being trapped in the In_2_S_3_ layer, the interfacial dynamics in an Al_2_O_3_/In_2_S_3_/Cu_3_BiS_3_ control sample were interrogated by transient absorption spectroscopy. In this sample, no electron injection from the metal sulfides to Al_2_O_3_ is expected, due to the much higher conduction band energy of Al_2_O_3_ compared to TiO_2_. However, if a transient absorption signal is observed in this sample, it would most likely stem from separated electrons or holes in In_2_S_3_ or Cu_3_BiS_3_, which would indicate that electrons are trapped in the In_2_S_3_ interlayer. As can be seen in Figure 8a,b (blue curves), no signals are observed in the Al_2_O_3_/In_2_S_3_/Cu_3_BiS_3_ sample, therefore indicating that the signal obtained in the mp-TiO_2_/In_2_S_3_/Cu_3_BiS_3_ sample stems from the recombination of injected electrons in mp-TiO_2_ and holes in Cu_3_BiS_3_. Moreover, the lack of signal in this sample also indicates the charge carriers formed in the Cu_3_BiS_3_ layer recombine with a lifetime faster than the detection limit (>200 ns) of our TAS setup.

## 3 Conclusion

In this study, we introduced a facile precursor-based method to prepare Cu_3_BiS_3_ thin films with an orthorhombic crystal structure. The time-resolved combined GISAXS and GIWAXS study revealed that the Cu_3_BiS_3_ film formation is initiated by the creation of small amorphous seeds, which become crystalline and grow to a diameter of 4 nm with a very narrow size distribution. During further growth, the size distribution becomes broader and the nanocrystals start to agglomerate. At 230 °C, thin films with grain sizes of approximately 80–150 nm are obtained. The Cu_3_BiS_3_ films exhibit a direct band gap of 1.55 eV and an absorption coefficient up to 1 × 10^5^ cm^–1^. Moreover, this versatile precursor-based route can be used to prepare Cu_3_BiS_3_ films on mesoporous metal oxide scaffolds to form heterojunctions, which can be applied in metal sulfide sensitized solar cells in the future. Transient absorption spectroscopic measurements reveal that the charge separation yields, which are found to be only moderate in mp-TiO_2_/Cu_3_BiS_3_ heterojunctions, can be significantly improved by the introduction of an In_2_S_3_ interlayer, and the generated charges are comparably long-lived (half-value time of ∼10 μs). This interface modification most probably reduces trap states at the surface of Cu_3_BiS_3_ and can be highly valuable regarding the development of Cu_3_BiS_3_ into a highly efficient, solution-processable, and inexpensive solar cell material.

## 4 Experimental Section

### 4.1 Materials synthesis

#### 4.1.1 Synthesis of Copper and Bismuth Xanthates

Copper(I) *O*-2,2-dimethylpentan-3-yl dithiocarbonate and indium *O*-2,2-dimethylpentan-3-yl-dithiocarbonate were received from Aglycon KG, where they were synthesized according to a previously published procedure.^38^

Bismuth(III) tri(*O*-isopropyldithiocarbonate): Carbon disulfide (8.37 g, 0.1 mol, 1 equiv) was added dropwise to a mixture of isopropanol (6 g, 0.1 mol, 1 equiv) and potassium hydroxide (5.6 g, 0.1 mol, 1 equiv), while the solution was stirred in an ice bath. After 1 h of stirring, the solvent was filtered off using a Buchner funnel. The raw product was first washed with acetone and then recrystallized via the addition of diethyl ether to the solution in methanol. The light yellow crystals were dried overnight in a desiccator to obtain potassium isopropyl xanthate.

Concentrated hydrochloric acid was added dropwise into 25 mL of bismuth(III) nitrate pentahydrate (2.4 mmol, 1.16 g, 1 equiv) aqueous dispersion until the solution became clear. After that, this clear solution was added dropwise into 6 mL of potassium isopropyl xanthate (7.2 mmol, 1.46 g, 3 equiv) aqueous solution and stirred at room temperature for one hour. The resulting mixture was then vacuum filtered. The yellow solid was dissolved in a small amount of chloroform and recrystallized via adding methanol. The bismuth isopropyl xanthate product was dried overnight in a desiccator. ^1^H NMR (400 MHz, CDCl_3_, δ): 5.65–5.83 (m, 1H, CH), 1.45–1.55 (d, 6H, 2 × CH_3_) ppm. Elemental anal. for BiS_6_O_3_C_12_H_21_: C 23.45, H 3.44; found C 23.56, H 3.35.

#### 4.1.2 Preparation of Copper Bismuth Sulfide Thin Films

The precursor solutions for the Cu_3_BiS_3_ films were prepared by dissolving copper(I) *O*-2,2-dimethylpentan-3-yl dithiocarbonate and bismuth(III) tri(*O*-isopropyldithiocarbonate) separately in chlorobenzene/pyridine (93/7, vol/vol, 0.18 mmol/mL), followed by mixing these solutions in a volume ratio of 3:1. The Cu_3_BiS_3_ thin films were formed on either bare glass substrates or glass substrates covered with mesoporous TiO_2_ or mesoporous Al_2_O_3_ layers. Therefore, the precursor solution was spin coated on the respective substrates and heated on a hot plate to 300 °C for 15 min in a glovebox (N_2_ atmosphere), whereby Cu_3_BiS_3_ is formed by thermal conversion of the metal xanthates. The In_2_S_3_ interlayers are prepared similarly to the Cu_3_BiS_3_ films by using indium(III) *O*-2,2-dimethylpentan-3-yl dithiocarbonate dissolved in chlorobenzene as the precursor solution. Typically, precursor solutions with a concentration of 0.18 mmol/mL were used.

Mesoporous TiO_2_ layers were prepared by spin-casting a TiO_2_ paste (30 NR-D, Greatcell Solar Materials) diluted with terpineol (1:2.5, w:w), and the mesoporous Al_2_O_3_ films were spin coated from a 20% (wt) dispersion in isopropanol on glass substrates. After spin coating, the films were dried for 5 min at 80 °C on a hot plate before they were sintered at 450 °C for 1 h in a furnace in ambient atmosphere.

### 4.2 Characterization Techniques

^1^H NMR spectra were recorded on a 400 MHz Bruker Avance spectrometer. Elemental analyses were carried out on a Universal CHNS elemental analyzer Vario El III. X-ray diffraction (XRD) patterns were measured on a PANalytical X’Pert Pro MRD diffractometer at 40 kV and 40 mA or a Rigaku Miniflex 600 with a D/Tex Ultra detector at 40 kV and 15 mA using Cu Kα radiation. Raman spectroscopy was performed on a LabRAM Infinity spectrometer (Horiba) using a 633 nm He–Ne laser. Scanning electron microscopy–electron dispersive X-ray (SEM-EDX) measurements were carried out on a JEOL 6400 scanning electron microscope operated at 20 kV. Scanning electron microscopic images were acquired on a LEO 1525 field emission scanning electron microscope operated at 5 kV using an In lens detector or a TESCAN MIRA3 field emission scanning electron microscope operated at 5 kV using an In-beam secondary electron detector. The samples for SEM characterizations were coated with chromium (5 nm) by sputtering before the measurements. XPS characterizations were performed on a Nexsa G2 XPS system (Thermo Fisher Scientific Inc.) using a monochromatized Al Kα X-ray source. The analyzer operated with a pass energy of 20 eV and a step size of 0.100 eV. The sample surface was cleaned by sputtering prior to the XPS investigations.

Transmittance and reflectance spectra for the determination of the optical absorption coefficient as well as the absorption spectra were recorded on a Shimadzu 2600 spectrophotometer equipped with an ISR-2600Plus integrating sphere attachment. The optical band gaps were estimated from (α*h*ν)^2^ vs *h*ν plots by extrapolating the linear part of the function. Layer thicknesses were measured using a Veeco Dektak surface profiler. The Cu_3_BiS_3_ film used for the determination of the absorption coefficient had a layer thickness of 65 nm.

2D grazing incidence small and wide angle X-ray scattering (GISAXS, GIWAXS) measurements were performed at the Austrian SAXS beamline 5.2L of the electron storage ring ELETTRA (Italy).^51^ During the temperature scan, data were recorded with a 6 s time resolution using a Pilatus 1M detector (Dectris). For detection of the GIWAXS signal, a Pilatus 100K detector from Dectris was used. The sample to detector distances have been adjusted to be able to access a *q*-range (*q* = 4π/λ·sin(2θ/2), 2θ represents the scattering angle) between 0.1 and 3.3 nm^–1^ with the GISAXS measurements and a 2θ range between 24 and 55° with the GIWAXS measurements. The measurements were performed at a photon energy of 8 keV. The samples were placed in a heating cell (DHS 1100 from Anton Paar GmbH, Graz, Austria) equipped with a custom-made dome with Kapton polyimide film windows and were heated from 30 °C up to 350 °C at a heating rate of 10 °C/min under a nitrogen atmosphere. The measurements were performed with an incidence angle of 0.25°. The angular calibration of the detectors was carried out using silver behenate powder (*d*-spacing of 58.38 Å) and *p*-bromobenzoic acid, respectively.

Microsecond transient absorption spectroscopy (μs-TAS) measurements were performed by exciting the samples in an inert atmosphere using a dye laser (Photon Technology International Inc. GL-301) pumped by a nitrogen laser (Photon Technology International Inc. GL-3300). A 100 W quartz halogen lamp (Bentham, IL 1) with a stabilized power supply (Bentham, 605) was used as the probe light source. A silicon photodiode (Hamamatsu Photonics, S1722-01) was used to detect the probe light passing through the sample, and the signal was amplified before being passed through electronic band-pass filters (Costronics Electronics). The amplified signal was collected with a digital oscilloscope (Tektronics, DPO3012), which was synchronized with a trigger signal from the pump laser pulse from a photodiode (Thorlabs Inc., DET210).

## Supporting Information Available

Additional images, GISAXS, XRD, SEM, SEM-EDX, XPS, and UV–vis data and information about the GISAXS data analysis (PDF)
Video S1 of the conversion of a spin coated precursor film to the Cu_3_BiS_3_ film (MP4)
Video S2 of the conversion of a drop coated precursor film to the Cu_3_BiS_3_ film (MP4)
