# Supplementary material for: Solution-Processable Cu3BiS3 Thin Films: Growth Process Insights and Increased Charge Generation Properties by Interface Modification
Source: ACS Appl Mater Interfaces. 2023 Aug 25;15(35):41624–33. doi: 10.1021/acsami.3c10297 (PMC10485802; doi:10.1021/acsami.3c10297)
Supplement: Supplementary file 1 — am3c10297_si_001.pdf [file am3c10297_si_001.pdf]

Supporting Information

Solution-processable Cu_3BiS_3 Thin Films: Growth Process Insights and Increased Charge Generation Properties by Interface Modification

Thomas Rath,[1,2] Jose M. Marin-Beloqui,[1] Xinyu Bai,[1] Astrid-Caroline Knall,[2] Marco Sigl,[2] Fernando G. Warchomicka,[3] Thomas Griesser,[4] Heinz Amenitsch,[5]* and Saif A. Haque[1]**

[1] Department of Chemistry, Imperial College London, Molecular Sciences Research Hub White City Campus, Wood Lane, W12 0BZ, UK

[2] Institute for Chemistry and Technology of Materials, NAWI Graz, Graz University of Technology, Stremayrgasse 9, 8010 Graz, Austria

[3] Institute of Materials Science, Joining and Forming, Graz University of Technology, Kopernikusgasse 24, 8010, Graz, Austria

[4] Institute of Chemistry of Polymeric Materials, Montanuniveristät Leoben, Otto Glöckelstrasse 2, 8700 Leoben, Austria

[5] Institute of Inorganic Chemistry, NAWI Graz, Graz University of Technology, Stremayrgasse 9, 8010 Graz, Austria

* Corresponding Authors: T. Rath (thomas.rath@tugraz.at), H. Amenitsch (amenitsch@tugraz.at), S. A. Haque (s.a.haque@imperial.ac.uk)

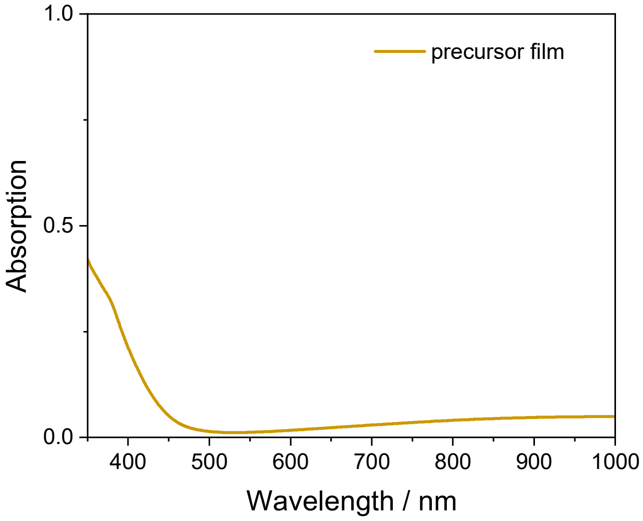

Figure S1. UV-Vis spectrum of a spin coated pale yellow precursor film consisting of copper and bismuth xanthates (molar ratio: 3:1).

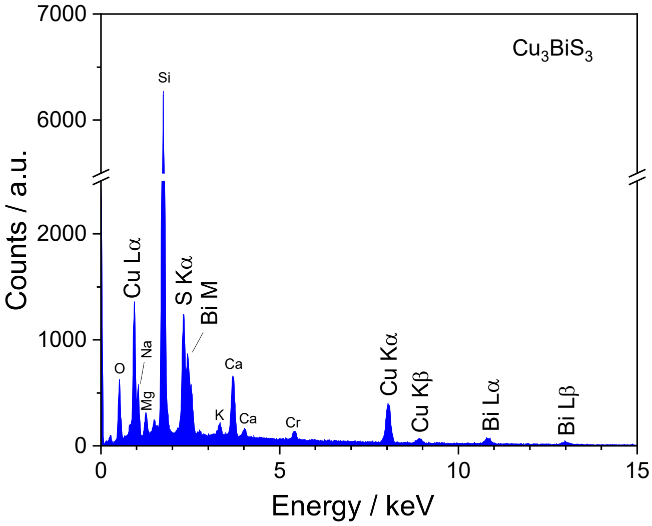

Figure S2. EDX spectrum of a C_3BiS_3 thin film on a glass substrate. In addition to the lines of Cu, Bi and S, the spectrum reveals the characteristic lines of the elements contained in the glass substrate as well as of Cr stemming from the Cr-coating of the sample applied to reduce electrical charging.

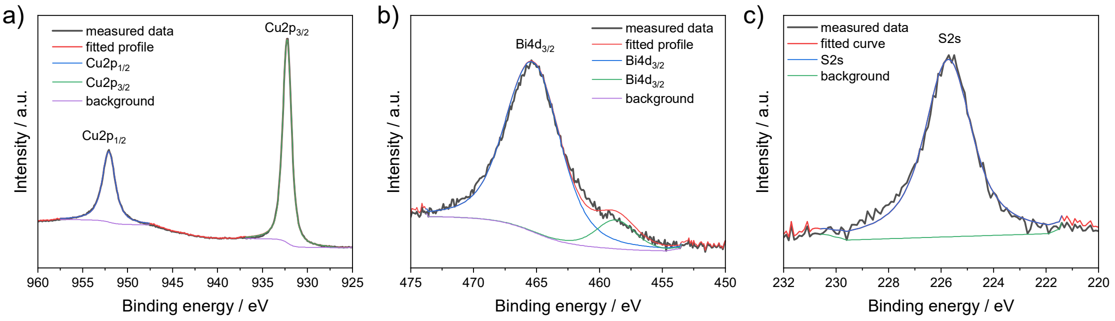

Figure S3. High resolution XPS spectra of a Cu_3BiS_3 film on a silicon substrate in the regions of (a) the Cu2p peaks, (b) the $Bi4d_{3/2}$ peak and (c) the S2s peak.

Determination of the Porod invariant from the GISAXS data:

As a sensitive estimate of changes related to the mass as well as the shape of the scattering pattern, the Porod invariant Q has been calculated by integrating the in-plane GISAXS scattering patterns over the q-range from 0.1 and 1.3 nm^{-1} with equation 1:

$$Q = \int_{q_{min}}^{q_{max}} dq_h \; {q_h}^2 \, I(q_h) \qquad (1)$$

$I(q_h)$ denotes the scattering pattern (in-plane), q_{min} / q_{max} the minimum and maximum q-values and q_h the q-axis (in-plane).

Fitting of the GISAXS data:

To extract characteristic parameters of the nanocrystalline Cu_3BiS_3 films during their formation, the GISAXS data were fitted with the following function (equation 2):

$$I_{calc}(q) = I_0 * F(q) * S_{SHS}(q) + I_{Porod}(q) + BG \qquad (2)$$

In this equation, I_0 is an intensity scalar, $F(q)$ stands for the form factor scattering, $S_{SHS}(q)$ denotes the structure factor contribution, $I_{Porod}(q)$ represents the Porod-contribution stemming from larger aggregates and BG is a constant background.

For the description of the form factor scattering, the function uses the model distributed spheres, which number weighted size distribution $D_N(r)$ is given by a Schulz distribution with R_S and

σ_{rat} as the mean and the polydispersity index (sigma/mean value). The volume weighted size distribution $D_V(r)$ has been calculated by the number weighted size distribution $D_N(r)$ with the corresponding factor of r^3.

The structure factor contribution is described by a sticky hard sphere model. The used parameters are illustrated in the scheme in Figure S4. Further details about the fitting function are given in Refs. 1-5.

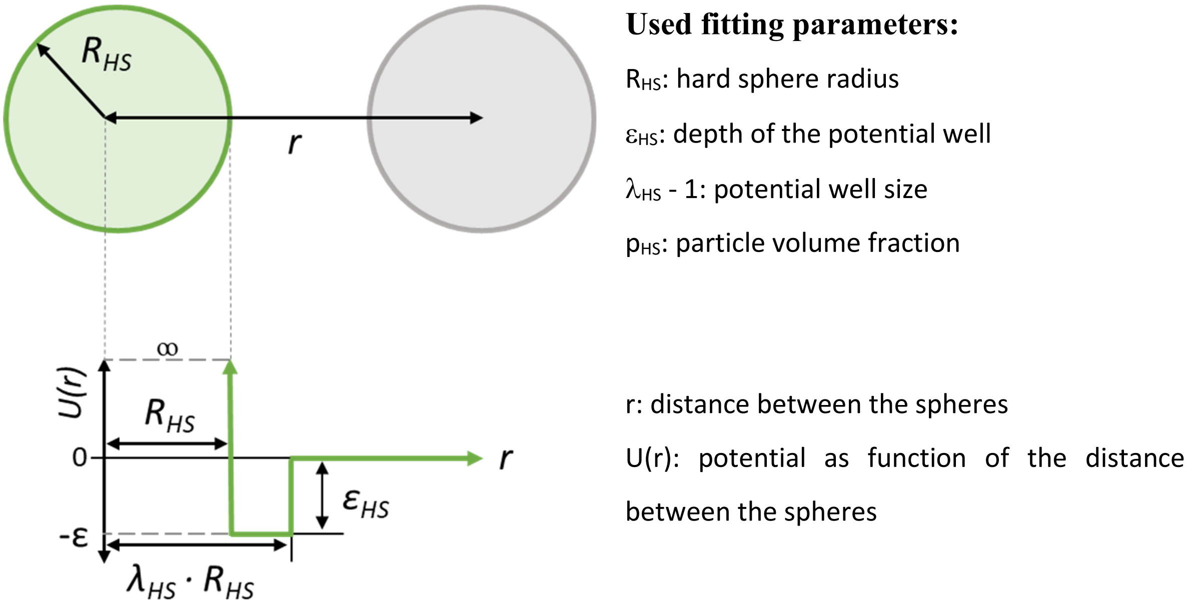

Figure S4. Schematic illustration of the used sticky hard sphere structure factor model and the respective fitting parameters.

The scattering of large-scale aggregates with dimensions beyond the resolution limit of SAXS are described using a power law based on equation 3, which is described in Ref. 6:

$$I_{Porod}(q) = c_p * q^{-e} \qquad (3)$$

with c_p the power law constant and e the power law exponent (Note: for e=4, it represents the Porod law). The Porod area was obtained by integrating the equation above over the q-range from 0.1 and 1.3 nm^{-1}.

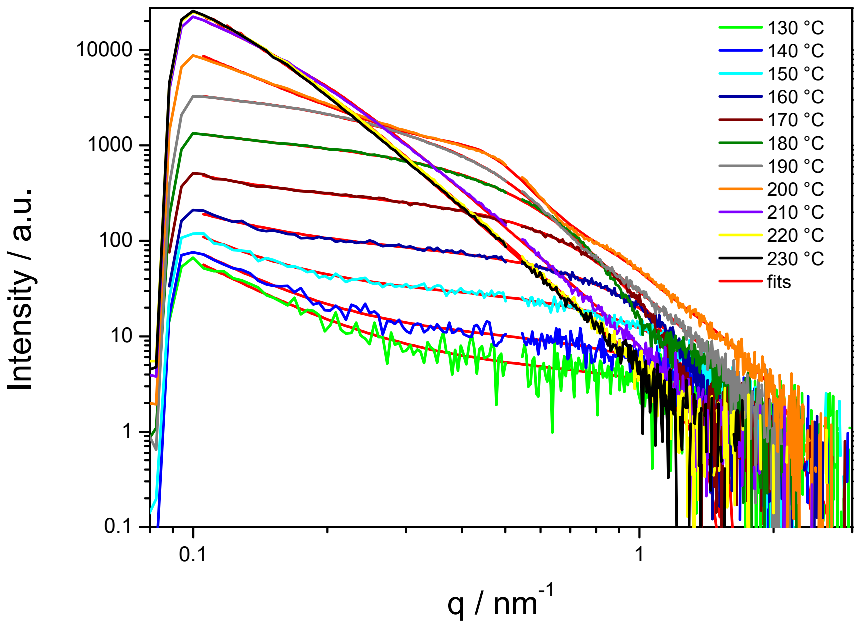

Figure S5. GISAXS curves (in-plane) at different temperatures and the corresponding fits.

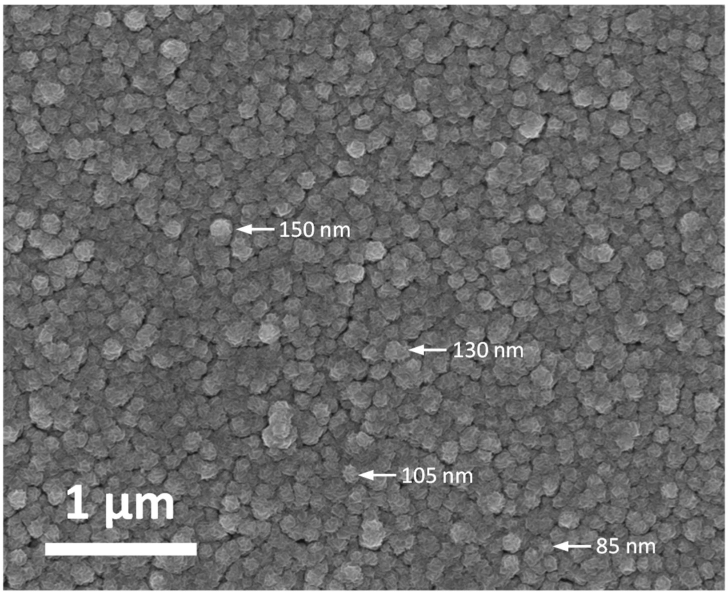

Figure S6. SEM image of a Cu_3BiS_3 film on glass prepared via spin coating of the precursor solution and subsequent annealing at 350 °C. The particle sizes of the nanocrystals in the film range between 80 and 150 nm.

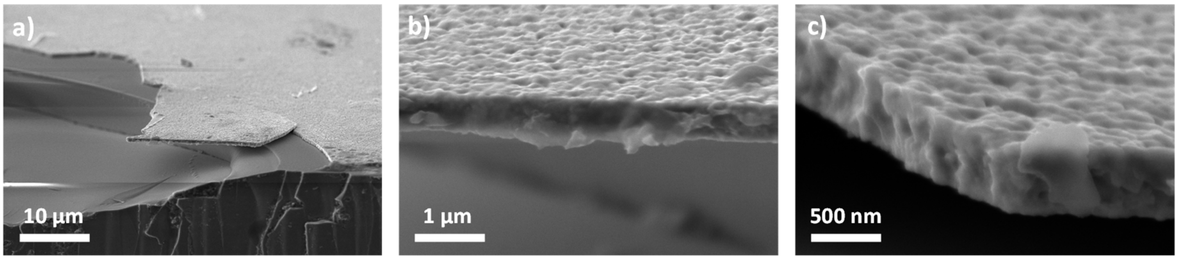

Figure S7. Cross section SEM images in different magnifications (a - c) of an approx. 500 nm thick Cu_3BiS_3 film prepared via drop coating of the precursor layer and annealing at 350 °C.

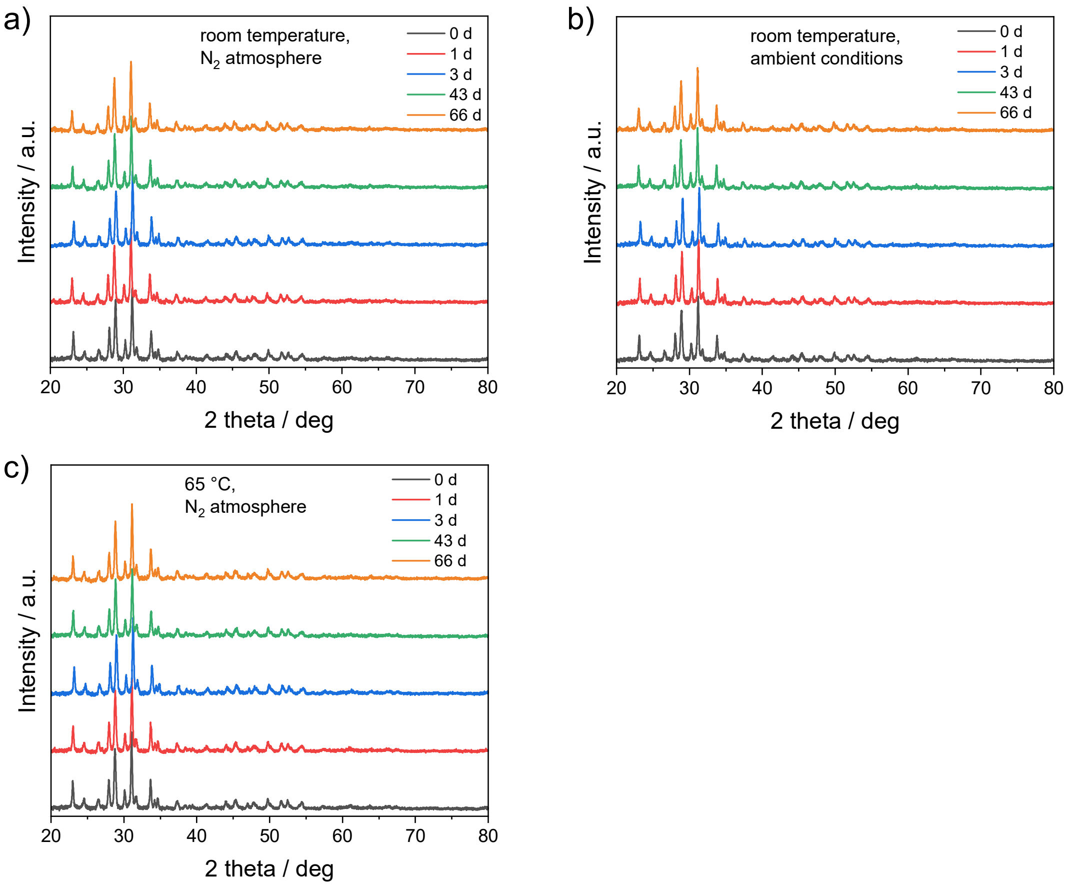

Figure S8. XRD patterns of Cu_3BiS_3 thin films at different times (0 to 66 days) during (a) storage at room temperature in inert atmosphere, (b) storage at room temperature in ambient conditions and (c) storage at 65 °C in inert atmosphere.

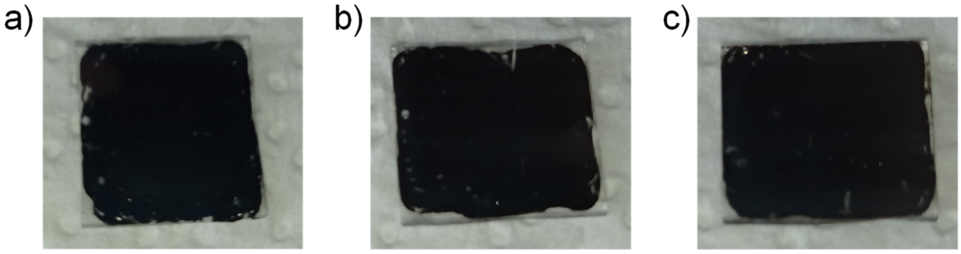

Figure S9. Pictures of the Cu_3BiS_3 samples after the stability investigations; (a) after storage at room temperature in inert atmosphere, (b) after storage at room temperature in ambient conditions and (c) after storage at 65 °C in inert atmosphere.

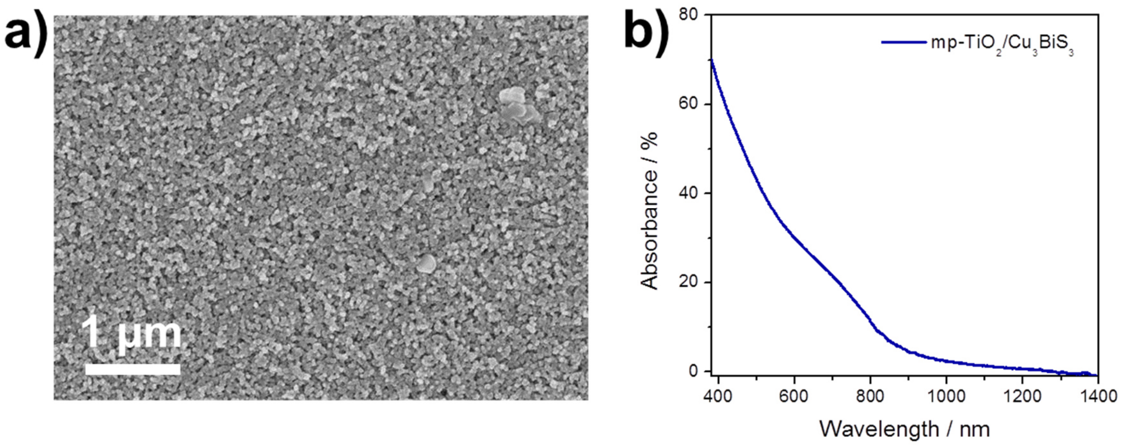

Figure S10. (a) Top view SEM image and (b) UV-Vis spectrum of a mp-TiO_2/Cu_3BiS_3 film.

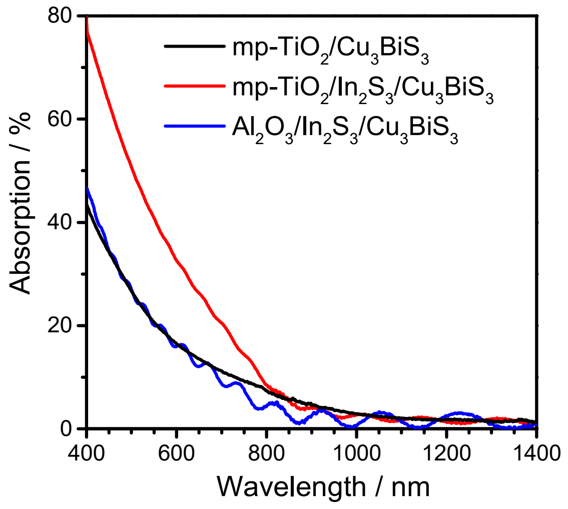

Figure S11. UV-Vis absorption spectra of the samples used for the transient absorption measurements.

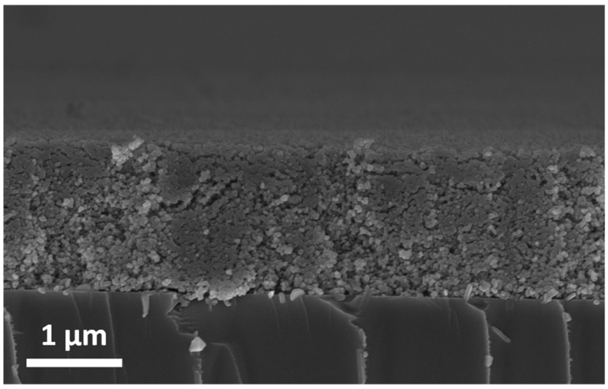

Figure S12. Cross section SEM image of a mp-TiO_2/In_2S_3/Cu_3BiS_3 sample.

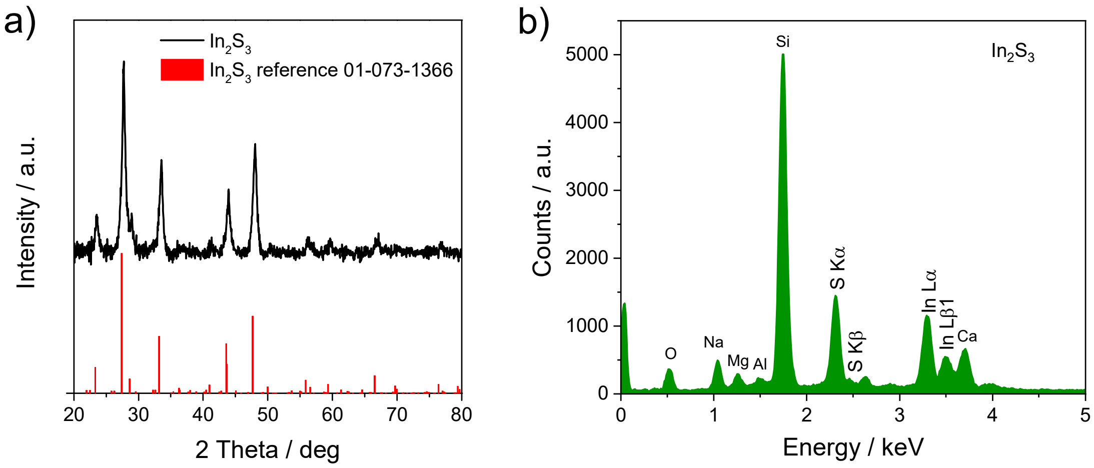

Figure S13. (a) X-ray diffraction pattern and (b) SEM-EDX spectrum of an In_2S_3 thin film prepared from indium 2,2-dimethylpentyl xanthate as precursor on a glass substrate. In addition to the lines of In and S, the EDX-spectrum reveals the characteristic lines of the elements contained in the glass substrate.

References:

[1] Rigodanza, F.; Burian, M.; Arcudi, F.; Dordevic, L.; Amenitsch, H.; Prato, M. Snapshots into Carbon Dots Formation Through a Combined Spectroscopic Approach, *Nat. Commun.*, **2021**, *12*, 2640.

[2] Kotlarchyk, M.; Chen, S. H. Analysis of Small Angle Neutron Scattering Spectra from Polydisperse Interacting Colloids, *J. Chem. Phys.,* **1983**, *79*, 2461–2469.

[3] Kotlarchyk, M.; Stephens, R. B.; Huang, J. S. Study of Schultz Distribution to Model Polydispersity of Microemulsion Droplets, *J. Phys. Chem.,* **1988**, *92*, 1533–1538.

[4] Sharma, R. V.; Sharma, K. C. The Structure Factor and the Transport Properties of Dense Fluids Having Molecules with Square Well Potential, a Possible Generalization, *Phys. A Stat. Mech. Appl.,* **1977**, *89*, 213–218.

[5] Pontoni, D.; Finet, S.; Narayanan, T.; Rennie, A. R. Interactions and Kinetic Arrest in an Adhesive Hard-Sphere Colloidal System, *J. Chem. Phys*., **2003**, *119*, 6157–6165.

[6] Glatter, O.; Kratky, O.; editors. Small Angle X-Ray Scattering. London: *Academic Press Inc.*; **1982**. 515 p. ISBN: 0-12-286280-5.
